# Unusual paranasal sinus solitary neurofibroma and literature review

**DOI:** 10.1259/bjrcr.20230025

**Published:** 2023-09-12

**Authors:** Xian Chun Yang, Li Chen

**Affiliations:** 1 Medical College of Wuhan University of science and technology, Department of Radiology, Hanyang Hospital Affiliated to Wuhan University of science and technology, WuHan, China

## Abstract

Neurofibroma (NF) is a common benign peripheral neurogenic tumor that is rarely encountered in the paranasal sinus tract. In this report, we present a 55-year-old female who serendipitously discovered a maxillary sinus NF during a medical examination for a pulmonary nodule. The purpose of this article is to enhance medical practitioners' comprehension of paranasal sinus solitary NF by exploring cases, summarizing occurrences located in the paranasal sinus tract and conducting an organized review of paranasal sinus tract NF.

## Case presentation

The patient was a 55-year-old female. Four weeks prior, she had presented with left neck pain and palpable mass similar to that of a broad bean, unaccompanied by local redness or swelling. She was also accompanied by cough, scanty white sputum, no fever, no sore throat, no chest pain, and no chest tightness or discomfort.

## Investigations

**Figure 1.**
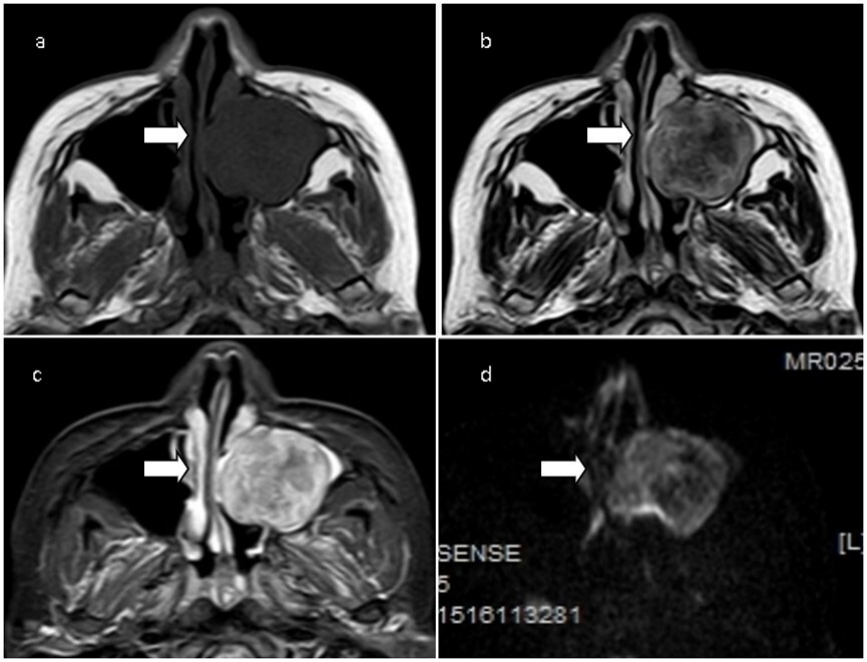
Transverse MR imaging of the nasal sinus demonstrated a solid mass occupying her left maxillary sinus(white arrows), measuring approximately 45 mm × 36 mm × 26 mm, and exhibiting hypointensity on T1WI(a), inhomogeneous hyperintensity on T2WI(b) and fat-suppression sequence(c), and moderate hyperintensity on DWI(d).

**Figure 2.**
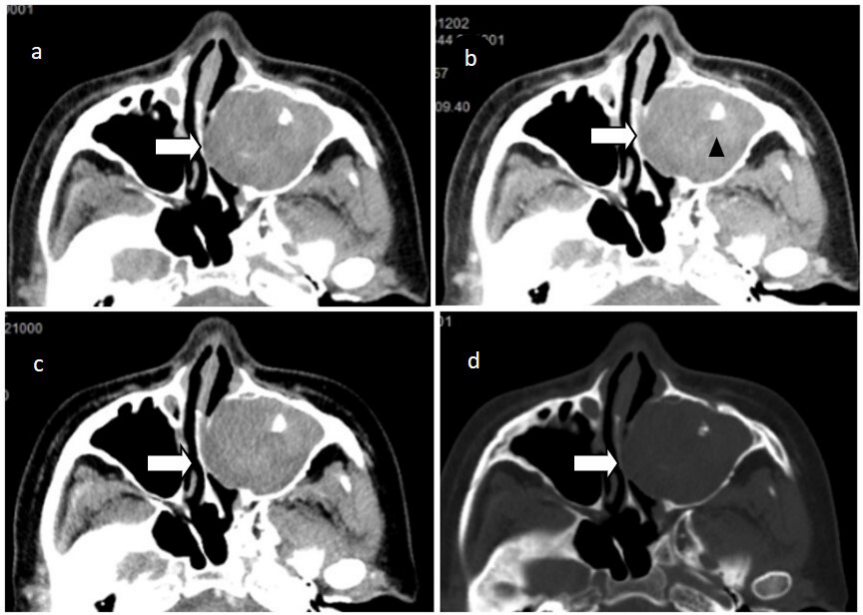
Axial non contrast-enhanced CT imaging(a) showed a round solid soft tissue mass(white arrows) in the left maxillary sinus with punctate calcification at its center(black arrow head). Contrast enhanced CT imaging(c and d) indicated mild uneven enhancement of the mass (an increase of about 5-10 HU); her maxillary sinus cavity was distended with bone resorption on its inner wall(d).

At first, the patient was diagnosed with cervical lymphadenopathy via outpatient ultrasound, and then a chest CT scan at our hospital revealed a tumor lesion in the left lower lobe. Subsequently, non-contrast-enhanced MRI of the brain (Figure 1) showed a solid mass in her left maxillary sinus measuring approximately 45 × 36 mm × 26 mm. The lesion exhibited expansive growth along the left maxillary sinus and signs of bone resorption on its medial wall. CT enhancement scan of the nasal sinus demonstrated a solid mass in the left maxillary sinus exhibiting slight delayed enhancement (Figure 2).

## Treatment/Outcome/Follow-up

Subsequently, biopsy of cervical lymph nodes indicated metastatic adenocarcinoma, thus she was diagnosed with lung adenocarcinoma and lymph node metastasis, presaging her poor prognosis.In accordance with imaging diagnostics, the nasal sinus mass was thought to be benign. A dialogue with the patient regarding further management ensued, eventually resulting in her consent for priority surgery of maxillary sinus masses. Postoperative complications were not observed. The patient was discharged ten days after surgery. An MRI scan conducted five months post-surgery revealed no recurrence. For the treatment of lung cancer, the patient underwent gene detection and targeted chemotherapy successively; however, the latest follow-up displayed lacklustre efficacy and CT displayed metastases of liver, lung and spine.

## Pathological findings

**Figure 3.**
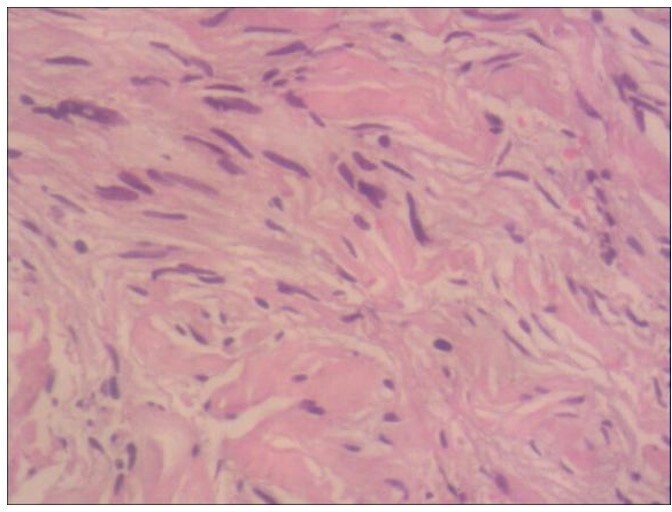
Pathological section microscopy showed abundant tumor cells presenting multiple elongated spindle cells scattered in Wagner Meissner body (HE×100).

Since the nasal sinus mass may have been benign, endoscopic resection of the left maxillary sinus mass was performed immediately; pathological results indicated paranasal sinus tract neurofibroma (NF) (Figure 3).

## Discussion

NF is a common benign neurogenic tumor, which rarely occurs in the paranasal sinus.^1^ So far, fewer than 100 cases have been reported in the literature around the world, mostly as individual case reports. It is mainly seen in individuals between 10 and 70 years old, with a peak age of onset at 30 years old; it is slightly more common in females than males.^2,3^ Most paranasal sinus NFs are primary solitary lesions (90%), with only a few related to type I neurofibrosis (10%).^4,5^

A case of paranasal sinus NF in the maxillary sinus is reported here. Although she has lung adenocarcinoma with cervical lymph node metastasis, there is no evidence of association among them.

Only 15 cases of NFs involving paranasal sinuses were found by searching literature and summarised in Table 1.^2,3,5–17^ Of these cases, three were related to neurofibromatosis type I and the other 12 were primary solitary NFs. Including this case reported here, there was a 1:2 male-to-female ratio among these 16 reports; onset age ranged from 10 to 79 years old with a median age of 45 years old.

**Table 1.** Cases of paranasal sinus neurofibroma

Publication date	Author	Sex	Age	Sym.	Loc	Exa.	M	BD	SS	CA	N/C	SE	SI
1975^6^	YVON Robitaille	F	45	*A* + C+F	①	X-ray	U	N	U	U	U	U	U
1979^7^	M.K. Agarwal	M	45	*A* + B+D	①	X-ray	U	Y	N	U	U	U	U
1988^8^	D.J. Stevens	41	F	*B* + G	④	CT	Y	Y	Y	N	N	U	Y
1988^8^	D.J. Stevens	F	49	*C* + G	⑤	X-ray+CT	Y	Y	Y	N	N	U	N
2004^9^	A.R. Mondal	M	38	*A* + C	①	CT	Y	Y	Y	N	U	U	Y
2005^10^	Carsten Christof Boedeker	M	25	*A* + F+G	②	CT + MRI	Y	Y	N	N	N	U	N
2009^11^	E.E.M. Nao	F	35	*A* + G	②	CT	Y	Y	Y	N	N	U	Y
2010^12^	Maria F. Cegarra-Navarro	F	70	G	②	CT + MRI+C	Y	Y	Y	Y	N	Y	N
2010^5^	Ralph N. Abi Hachem	F	10	*C* + F	①	CT + MRI+C	Y	N	Y	N	N	Y	N
2012^13^	Sudhir B. Sharma	M	28	*A* + C	⑥	CT	Y	Y	Y	N	N	U	Y
2012^14^	Józef Komorski	F	79	*A* + B+G	②	CT + C	Y	Y	Y	N	Y	Y	Y
2011^15^	Satoshi Rokutanda	M	41	G	①	X-ray+CT	Y	N	N	N	N	U	N
2014^16^	Hongxing Li	F	26	*A* + C	⑥	CT + MRI+C	Y	Y	N	N	N	Y	Y
2014^2^	Deepali Jain	F	60	*A* + G	①	X-ray	U	N	Y	U	U	U	U
2016^4^	Darren Yap	M	70	G	①	MRI + C	Y	Y	Y	U	N	Y	Y

⑥, left sphenoid sinus; ④, left ethmoid sinus; ②, left maxillary sinus; ③, right ethmoid sinus; ⑤, right frontal sinus; ①, right maxillary sinus; A, swelling; B, nasal congestion; BD, bone destruction; C, pain; CA, calcification; D, epistaxis; E, runny nose; Examination, EXa.; F, skin changes; G, others (Including sensory abnormalities, facial discomfort, dysphagia, eye abnormalities, tooth abnormalities, abnormal occlusal function, etc); M, mass; N, NO; N/S, necrosis / cysts; SE, significantly enhanced; SI, surrounding infiltration; SS, sinus swelling; U, Unknown; Y, YES.

Clinical manifestations of sinus NF lack specificity and vary depending on location. Common clinical manifestations include local swelling, pain, nasal congestion or runny nose as well as epistaxis or facial discomfort; occasionally invading the orbit can lead to exophthalmos and visual impairment,^4^ invading alveolar bone can lead to tooth loosening or abnormal occlusion,^2^ and invading brain tissue can cause headaches.^17^ Solitary NFs differ from NF1 related ones due to lack of common skin coffee spots or family history associated with them.^5^

The imaging findings for paranasal sinus NFs show nonspecific changes but understanding certain characteristics may help prompt diagnosis: (1) Paranasal sinus NFs is most commonly found in maxillary sinuses followed by frontal ones while ethmoid and sphenoid are rarer; out of 20 cases summarised here 13 were located in maxillary while four were located at frontal ones and one at ethmoid; other cases involved both ethmoid and sphenoid but all part of nasal NF. (2) Paranasal sinus NFs are more commonly seen as solitary lesions than NF1-related ones; only 3 of 16 cases (18.75%) being NF1-related. Whereas 90% being solitary lesions according to Azani et al,^3^ which was consistent with our findings. NF1-associated tumors often present multiple lesions involving multiple sites simultaneously. (3) On X-ray paranasal sinus, NFs usually manifest by decreased transparency within affected area sometimes accompanied by dilated Sinuses. Whereas CT usually shows heterogeneous solid mass inside nasal cavity, sometimes cystic too, when small tumor size does not change size within affected area; however, large tumors may expand said cavity or even cause local bone absorption/breakage through its walls into orbital/intracranial/pterygomaxillary fossa areas, respectively; contrast enhancement showed mild up to obvious enhancement which may be linked toward tissue components within said tumor.^12,18^ While MRI showed medium low signal on T1WI, heterogeneous medium signal/high signal on T2WI, without limited diffusion on DWI.^18^ (4) Despite invasive nature potentially leading up toward malignant NF recurrence after surgical resection almost non-existent.^12,19^

## Differential diagnosis

The differential diagnosis of paranasal sinus NFs includes cysts, schwannomas, solitary fibroid tumors, inflammatory pseudotumors, fungal granulomas, inverted papillomas, ectopic meningiomas, ossifying fibroids, leiomyosarcomas, lymphomas, metastasis and so on. If low density or cystic components are found inside the tumor, maxillary sinus cysts/schwannomas should be differentiated. When solid tumors with delayed enhancement are manifested, solitary fibromas, inflammatory pseudotumors, fungal granulomas, inverted papillomas, and ectopic meningiomas need be distinguished, while calcification is detected, ossifying fibromas would be differentiated first. Once bone walls and surrounding tissues are invaded, leiomyosarcoma, lymphoma, and metastatic tumor should be identified.

## Treatment

Surgery is currently the main treatment option for paranasal sinus NFs. Although slow progression and good prognosis are common, radiotherapy and chemotherapy may be performed while malignant lesions are detected.^18^

In conclusion, paranasal sinus NFs is a rare benign neurogenic neoplasms, which expressed either isolation or NF1 correlation. Despite there is non-specific in-clinical presentation and imaging features of it, consideration should be given to the possibility of paranasal sinus NFs when diagnosed benign masses and solid tumors of the paranasal sinus by imaging, especially in those with NF1.

## Learning points

1. Paranasal sinus solitary NFs are a rare type of tumor mainly occurring within the maxillary sinuses.
2. The clinical manifestations of paranasal sinus solitary NF are nonspecific in nature. Commonly observed symptoms include localised swelling, pain, nasal obstruction, rhinorrhea, and epistaxis.
3. When imaging reveals a heterogeneous solid mass in the paranasal sinus accompanied by mild delayed enhancement or obvious enhancement, consideration should be given to the potentiality of NF, regardless of the presence of bone destruction.
4. The imaging manifestations of paranasal sinus NFs are non-specific making it difficult to differentiate from other conditions. The diagnosis primarily relies on tissue biopsy.
